# Kidney Cysts in Children With Alport Syndrome: A Report of 3 Cases

**DOI:** 10.1016/j.xkme.2024.100815

**Published:** 2024-03-25

**Authors:** Yeun-Wen Chang, Daw-Yang Hwang, Tung-Ying Chen, Chun-Chen Lin, Min-Hua Tseng, Jeng-Daw Tsai

**Affiliations:** 1 Department of Pediatrics, Taipei Tzu Chi Hospital, Buddhist Tzu Chi Medical Foundation, New Taipei City, Taiwan; 2 Division of Nephrology, Department of Pediatrics, MacKay Children’s Hospital, Taipei, Taiwan; 3 National Institute of Cancer Research, National Health Research Institutes, Tainan, Taiwan; 4 Division of Nephrology, Department of Medicine, Kaohsiung Medical University Hospital, Kaohsiung Medical University, Kaohsiung, Taiwan; 5 Department of Pathology, MacKay Memorial Hospital, Taipei, Taiwan; 6 Department of Medicine, MacKay Medical College, New Taipei City, Taiwan; 7 MacKay Junior College of Medicine, Nursing and Management, Taipei, Taiwan; 8 Division of Nephrology, Department of Pediatrics, Chang Gung Memorial Hospital and Chang Gung University, Taoyuan, Taiwan

**Keywords:** Alport syndrome, pathogenic *COL4A3-COL4A5* variant, kidney cysts, autosomal-dominant polycystic kidney disease (ADPKD), kidney failure

## Abstract

Alport syndrome (AS) is a progressive hereditary kidney disease characterized by hematuria, proteinuria, and progressive kidney dysfunction accompanied by sensorineural hearing loss and ocular abnormalities. Pathogenic *COL4A3-5* variants can result in different AS spectra. Further, kidney cysts have been reported in adults with AS. However, the relationship between kidney cysts and AS remains unclear. Here, we report 3 cases of AS in children that occurred with kidney cysts. The patient in case 1 was initially diagnosed with IgA nephropathy at the age of 8 years but later developed bilateral multiple kidney cysts at the age of 17 years, suggesting autosomal-dominant polycystic kidney disease. Whole-exome sequencing identified a pathogenic *COL4A5* variant and confirmed the AS diagnosis. The patients in cases 2 and 3 had already been diagnosed with X-linked AS using kidney biopsy and genetic analysis. Initial kidney ultrasonography showed nephromegaly; however, kidney cyst formation was observed during their annual follow-up. Our study supports the association between AS and kidney cysts. Kidney cysts in adolescents with suspected AS should not discourage clinicians from testing for pathogenic *COL4A3-COL4A5* variants. Early detection of kidney cysts is critical because it may indicate kidney disease progression.

## Introduction

Alport syndrome (AS) typically presents with hematuria, proteinuria, and progressive kidney failure, often accompanied by sensorineural hearing impairment and ocular problems.^1^ It is caused by pathogenic *COL4A3-5* variants encoding the α3-5 chains of collagen type IV of the basement membranes. A recent classification scheme categorizes *COL4A3-5* diseases into X-linked, autosomal, and digenic AS.^1^ X-linked inheritance is caused by pathogenic *COL4A5* variants. Hemizygous male and female patients have 100% and 25% risk of progression to kidney failure, respectively.^1^

With the broad implementation of next-generation sequencing, the spectrum of kidney phenotypes associated with pathogenic *COL4A3-5* variants has widened. The expanded phenotypic spectrum of AS may present with focal segmental glomerulosclerosis, idiopathic chronic kidney disease, steroid-resistant nephrotic syndrome, familial IgA nephropathy, and kidney cysts.^2^ Kidney cysts have been reported in adults with pathogenic *COL4A3-5* variants.^3^, ^4^, ^5^, ^6^ We report 3 cases of X-linked AS in children manifesting with kidney cysts since adolescence.

This study was conducted according to the guidelines of the Declaration of Helsinki and approved by the Institutional Review Board of MacKay Memorial Hospital (approval no.: 23MMHIS088e; April 20, 2023). Following the ethics review, the hospital review board affirmed the ethical compliance of the study and authorized the publication of the findings.

## Case Reports

### Case 1

A 7-year-old girl with a 4-year history of hematuria and a paternal history of gross hematuria was referred for further management. Work-up showed microscopic hematuria with proteinuria (urine protein-to-creatinine ratio: 0.9 mg/mg). Screening for secondary glomerulonephritis yielded negative results, and no hearing or visual abnormalities were observed. Kidney ultrasonography revealed increased cortical echogenicity. Captopril was prescribed.

**Figure 1:**
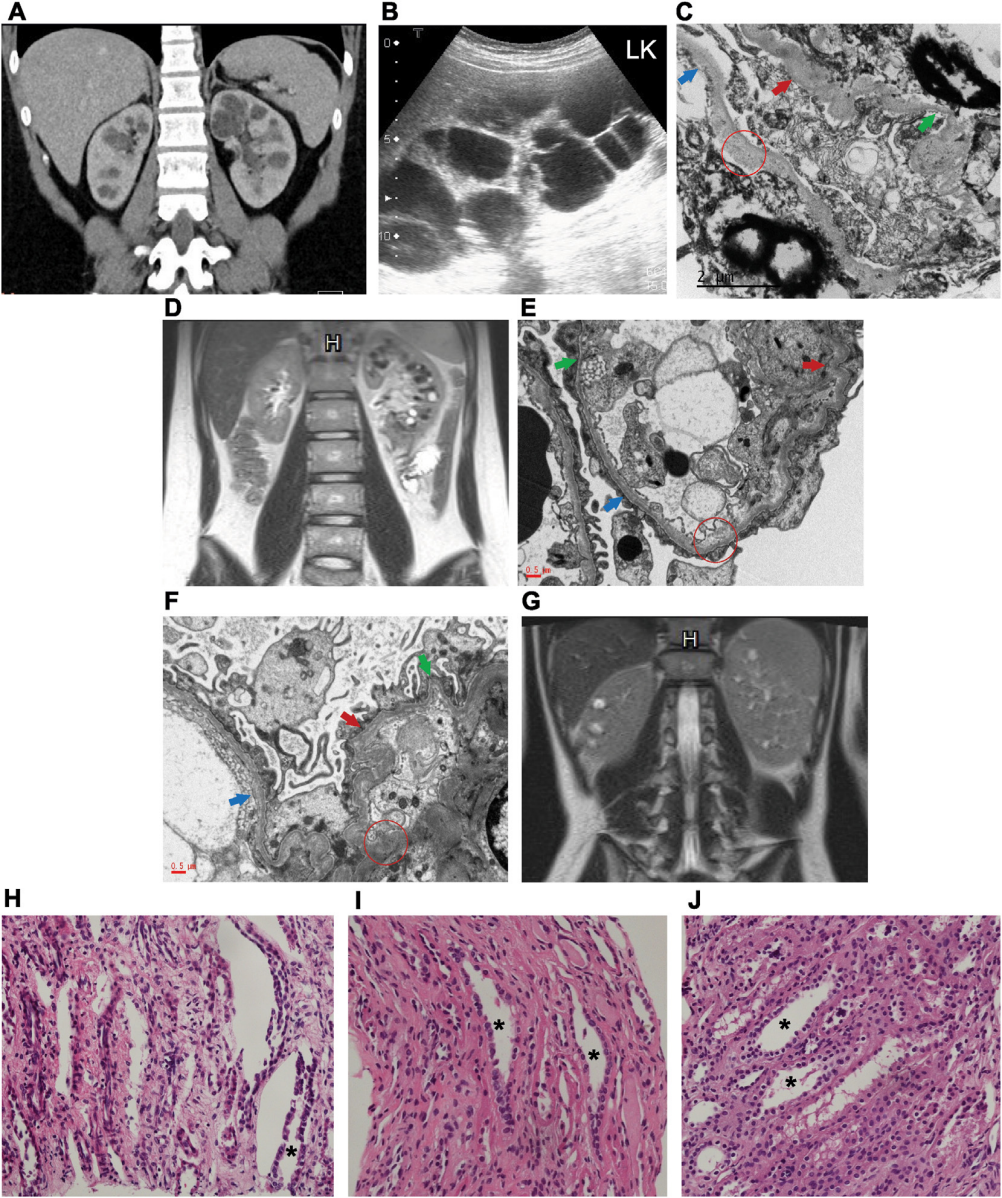
An image of kidney cysts and pathologic features of the kidney in Alport syndrome. (A) Case 1: a computed tomography image revealing multiple large cysts in the bilateral medullary area of the kidney. (B) Case 1: an ultrasonography image showing multiple cystic holes in renal parenchyma with a markedly enlarged kidney, mimicking autosomal-dominant polycystic kidney disease. (C) Case 1: an EM showing lamination (blue arrow), microparticles (red circle), irregular thinning (green arrow), and thickening (red arrow) of the GBM. (D) Case 2: a magnetic resonance image showing left nephromegaly with multiple medullary cysts. (E) Case 2: an EM showing lamination (blue arrow), microparticles (red circle), irregular thinning (green arrow), and thickening (red arrow) of the GBM. (F) Case 3: an EM showing lamination (blue arrow), microparticles (red circle), irregular thinning (green arrow), and thickening (red arrow) of the GBM. (G) Case 3: a magnetic resonance image revealing multiple bilateral medullary cysts. (H-J) Cases 1-3: light microscopy images showing dilatation of the distal tubules (∗); hematoxylin-eosin staining, ×200. Abbreviations: EM, electron micrograph; GBM, glomerular basement membrane.

At 8 years, a kidney biopsy was performed owing to the gradually progressive proteinuria (urine protein-to-creatinine ratio: 1.6 mg/mg). Light microscopy showed mesangial hypercellularity. Immunofluorescence microscopy was not conducted because of inadequate specimens. Electron microscopy revealed an inadequately fixed specimen with no electron-dense deposits in the glomeruli. Familial IgA nephropathy was suspected, and corticosteroids and azathioprine were administered for 2 years. The treatment response was poor, and proteinuria persisted. She was lost to follow-up and discontinued medication for 5 years. At 17 years, ultrasonography and computed tomography revealed multiple large cysts in the bilateral medullary areas of the kidney (Fig 1A), with a creatinine level of 0.9 mg/dL. Captopril was continuously administered. The creatinine level gradually increased to 1.7 mg/dL over the next 10 years. Progressive enlargement of the cysts and kidneys (approximately 18 cm) in the cortex and medulla was examined using subsequent ultrasonography (Fig 1B). She visited another nephrologist who administered tolvaptan without genetic analysis under the impression of autosomal-dominant polycystic kidney disease (ADPKD).   However, within 9 months, her glomerular filtration rate declined rapidly from 42 mL/min/1.73 m² to 24 mL/min/1.73 m². Proteinuria was further exacerbated (urine protein-to-creatinine ratio: 4.0 mg/mg), and hypertension was observed. Whole-exome sequencing identified a pathogenic *COL4A5* variant, c.3511C>T (p.Gln1171Ter), confirming X-linked AS. No pathogenic *PKD1* or *PKD2* variants were found. A previous kidney biopsy sample was refixed and reevaluated. Electron microscopy revealed irregular thinning and thickening of the glomerular basement membrane (Fig 1C). At 28 years, the patient developed kidney failure.

### Case 2

A 19-year-old girl with a family history of X-linked AS was followed up at our department since birth. Her father had been undergoing dialysis treatment since young adulthood. At 5 years, the patient developed proteinuria, and captopril treatment was initiated. Ultrasonography revealed left nephromegaly at the age of 8 years and multiple medullary cysts in the left kidney during follow-up at the age of 15 years. Magnetic resonance imaging showed left nephromegaly and multiple medullary cysts (Fig 1D). A kidney biopsy revealed lamination of the glomerular basement membrane with crumbs and a thin segmental basement membrane (Fig 1E). Whole-exome sequencing identified COL4A5:c.716G>T (p.Gly239Val) in the patient, her father, and sister. Her 21-year-old sister had persistent microscopic hematuria without proteinuria; kidney ultrasonography revealed normal-size kidneys without any cysts. The proteinuria remained stable (urine protein-to-creatinine ratio: 0.3-0.6 mg/mg), although her creatinine level gradually increased to 1.2 mg/dL, 6 years after increasing cyst development. No hearing or visual abnormalities are present currently. She is undergoing regular medical surveillance at an outpatient clinic.

### Case 3

An 8-year-old boy diagnosed with minimal change disease in Vietnam was referred to our hospital for investigation of steroid-resistant nephrotic syndrome with hematuria. Ultrasonography revealed bilateral nephromegaly. Despite the initiation of prednisolone and azathioprine for suspected IgA nephropathy, recurrent gross hematuria and nephrotic-range proteinuria necessitated a second kidney biopsy, revealing focal segmental glomerulosclerosis. Electron microscopy showed multilamellation and a segmentally thin glomerular basement membrane; thus, AS was suspected (Fig 1F). Whole-exome sequencing further revealed a COL4A5:exon 19-21 deletion, consistent with X-linked AS. Acoustic immittance and pure-tone audiometry revealed mild sensorineural hearing impairment. Ocular abnormalities or a family history of hematuria or kidney impairment was absent. A kidney cyst (7.6 × 9.2 mm) was detected during routine annual kidney ultrasonography at 14 years of age. Magnetic resonance imaging revealed multiple bilateral medullary cysts (Fig 1G).

## Discussion

Pathogenic *COL4A3-5* variants may account for focal segmental glomerulosclerosis, kidney failure, and familial IgA nephropathy in 30%, 10%, and 20% of cases, respectively.^2^ Furthermore, pathogenic *COL4A3-5* variants have been detected in adults with multiple kidney cysts.^3^, ^4^, ^5^, ^6^ The age of cyst onset is unknown. To the best of our knowledge, this is the first study to report that children with AS may develop kidney cysts during adolescence.

The incidence of and association between kidney cysts and AS remain unclear. Limited studies have reported AS with kidney cysts in adults.^3^, ^4^, ^5^, ^6^ Pierides et al^4^ first found multiple small or large kidney cysts in 4 older patients from 236 family members at risk for heterozygous pathogenic *COL4A3* variants. Sevillano et al^5^ found that 56% of patients with thin basement membrane disease who had hypertension, proteinuria, and chronic kidney disease had multiple kidney cysts, whereas those without proteinuria showed no cysts. The development of kidney cysts in thin basement membrane disease may predict unfavorable complications, including proteinuria, hypertension, and impaired kidney function.^5^ Gulati et al^3^ reported 6 cases of pathogenic type IV collagen variants among 18 individuals with multiple kidney cysts, either without pathogenic *PKD* variants or with a thin basement membrane disease diagnosis. Savige et al^6^ confirmed X-linked AS in a man with uremia who had bilateral kidney cysts of undetermined etiology.

Table 1 summarizes the reported clinical and genetic features of AS with kidney cysts, including our patients who are children. Cysts occur in both men and women with pathogenic *COL4A3-5* variants. Apart from our 3 patients, most patients with kidney cysts were aged older than 40 years.^3^, ^4^, ^5^, ^6^ Cysts always occur bilaterally in relatively large kidneys and vary in size and number. In our patients, most cysts were initially found in the medulla; however, they may affect both the cortex and medulla.^2^^,^^3^ Cysts may be detected in patients with normal or impaired kidney function. However, cyst formation usually follows pre-existing proteinuria.^4^^,^^5^ Furthermore, it often coexists with the focal segmental glomerulosclerosis pathology on biopsy, suggesting more severe basement membrane damage.^2^^,^^4^ In case 2, the patient had proteinuria at 5 years of age and developed cysts at 15 years of age. However, her sister, who had the same pathogenic variant, only experienced microscopic hematuria with no cysts. Cyst formation may indicate proteinuria and chronic kidney disease progression.^2^^,^^5^ Distinguishing AS from ADPKD is crucial; cysts in AS are smaller and fewer and do not usually distort the kidney outline or significantly increase the kidney volume.^2^^,^^6^ With progressive cystic enlargement, patient 1 was misdiagnosed with ADPKD. In case of difficulty in differentiating AS and ADPKD, genetic analysis can confirm the diagnosis and rule out the coincidence of both entities. Established proteinuria in ADPKD occurs late and is always associated with impaired kidney function and an extremely large kidney volume, differing significantly from the course of AS with kidney cysts.^7^

**Table 1:** A Summary of the Clinical and Genetic Features of Patients With a Documented Kidney Cyst in Alport Syndrome

	Age (y)/Sex (S) or Gender (G)^a^	Initial Diagnosis	SCr (mg/dL)	eGFR (mL/min/1.73 m^2^)/CKD Stage	Proteinuria	Kidney Size R/L (cm)	Kidney Cysts	Size of the Largest Cyst (mm)	Pathogenic Type IV Collagen Variant
**Pierides et al**^4^									
**Pedigrees 1**		FSGS, TBMD							
**Pedigrees 2**		FSGS, TBMD							COL4A3: c.G1334E
**Pedigrees 3**		FSGS, EM ND							COL4A3: c.G871C
**Pedigrees 4**		FSGS, EM ND							COL4A3: c.G871C
**Sevillano et al**^5^^,^^b^									
**Case 1**	54/F (G)	TBMD	0.5	136	0.43 (g/day)	11.6/11.2	2 in RK, 1 in LK	45	
**Case 2**	61/M (G)	TBMD	0.9	87	1.34 (g/day)	12/12	Multiple, bilateral	20	
**Case 3**	71/F (G)	TBMD	1.7	30	0.29 (g/day)	9/10	Multiple, bilateral	20	
**Case 4**	68/M (G)	TBMD	1.9	36	0.26 (g/day)	9.4/10	Multiple, bilateral	51	
**Case 5**	37/M (G)	TBMD	0.9	93	3.76 (g/day)	12/13	1 in RK, 10 in LK	30	
**Case 6**	66/F (G)	TBMD	0.5	108	0.34 (g/day)	12.2/12.8	Multiple, bilateral	10	
**Case 7**	64/M (G)	TBMD	3.1	21	0.32 (g/day)	10/10	4 in RK, 2 in LK	40	
**Sevillano et al**^5^**and Gulati et al**^3^^,^^c^									
**Case 8**	28/M (S)	TBMD		CKD stage 3	Yes	11.8/11.3	Multiple, bilateral	20	COL4A4: exon46:c.4503dupA
**Case 9**	42/M (S)	TBMD		CKD stage 3	Yes	16.7/17.0	Multiple, bilateral	70	COL4A4: exon24: c.1697– 1G>C
**Gulati et al**^3^^,^^c^									
**Case 10**	25/M (S)	NMD-ADPKD		125			Multiple, bilateral		COL4A4: exon28: c.G2383A
**Case 11**	52/F (S)	FSGS, TBMD		CKD stage 4		11.0/10.5	Multiple, bilateral		COL4A4: exon 39: c.3704delC
**Case 12**	49/F (S)	TBMD		CKD stage 2	No	10.5/10.9	Few, bilateral		COL4A5: exon16: c.C899T
**Savige et al**^6^									
**Case 13**	49/M (S)	Kidney failure, unknown cause	3.3	20	128.6 (mg/mmol)^e^		Multiple, bilateral	40	COL4A5: c.358G>A
**Present cases**^d^									
**Case 14**	17/F (S)	Familial IgA nephropathy	0.9	82	0.7 (mg/mg)^f^	18.6/16.0	Multiple, bilateral	24	COL4A5: c.3511C>T
**Case 15**	15/F (S)	AS	0.4	97	0.54 (mg/mg)^f^	9.8/12.9	Multiple, LK	10	COL4A5:c.716G>T
**Case 16**	14/M (S)	SRNS, FSGS	0.7	156	0.49 (mg/mg)^f^	10.3/10.7	4 in RK, 2 in LK	9.2	COL4A5: Exon 19-21 deletion

*Note*: Conversion factor for units: SCr in mg/dL to μmol/L, ×88.4.

Abbreviations: AS, Alport syndrome; CKD, chronic kidney disease; eGFR, estimated glomerular filtrate rate; EM, electron microscopy; F, female; FSGS, focal segmental glomerulosclerosis; G, Gender; LK, left kidney; M, male; ND, not done; NMD-ADPKD, no pathogenic PKD variant detected–autosomal-dominant polycystic kidney disease; NS, nephrotic syndrome; RK, right kidney; S, Sex; SCr, serum creatinine; SRNS, steroid-resistant nephrotic syndrome; TBMD, thin basement membrane disease.

a Sex refers to the biological characteristics that define humans as male or female. Gender refers to the socially constructed roles, behaviors, expressions, and identities that a society considers appropriate for males, females, and other genders.

b The clinical features of the cases were recorded at the end of the follow-up period.

c The ages of the cases were recorded at clinical/imaging or histopathologic diagnosis of ADPKD or TBMD.

d The clinical features of the cases were recorded at the time when kidney cysts were detected.

e Proteinuria (spot urine albumin-creatinine ratio).

f Proteinuria (spot urine protein-creatinine ratio).

Symptoms of X-linked AS are considered milder in women. However, approximately 25% of female patients with pathogenic *COL4A5* variants can have significant symptoms and progressive kidney dysfunction in old age.^1^ The 2 girls in this study exhibited persistent hematuria, proteinuria, and nephromegaly from early childhood before cyst development. Unexpectedly, patient 1 experienced progressive cystic enlargement and developed kidney failure at 28 years of age, suggesting that cyst formation could be a marker of early kidney function deterioration in women with X-linked AS. Further investigation into the causal relationship between cyst formation and kidney outcomes in patients with AS is warranted.

The pathogenesis of AS-associated kidney cysts remains unclear. Weakening of the basement membrane owing to abnormal type IV collagen in the distal tubule or glomerulus may induce or favor cyst formation. Dilated tubules and cystic dilatation of Bowman’s space observed in animal models, including a mouse model with pathogenic *COL4A4-5* variants and an autosomal-dominant AS dog model, support this hypothesis.^8^^,^^9^ In our cases, initial biopsy performed before cysts were evident on imaging did not reveal cysts. Instead, all patients showed distal tubule dilatation on pathology (Fig 1H, I, and J), and cysts were initially found in the medullary area. Whether distal tubule dilatation will eventually lead to cysts is unclear; however, we found that before the appearance of multiple kidney cysts on ultrasonography, the involved kidney usually becomes larger, like early ultrasound findings of ADPKD.^10^

In conclusion, although the cystic kidney phenotype may not be a predominant clinical manifestation of AS, our study strengthens the association between AS and kidney cysts. Kidney cysts in adolescents or adults with suspected AS should not discourage clinicians from testing for pathogenic *COL4A3-5* variants. Early detection of kidney cysts is vital because it may indicate kidney disease progression. Further studies are required to elucidate the pathogenesis of cyst formation and its influence on kidney outcomes.
